# Novel *IRF6* mutations in Chinese Han families with Van der Woude syndrome

**DOI:** 10.1002/mgg3.1196

**Published:** 2020-02-28

**Authors:** Yanqin Yu, Yatao Wan, Chuanqi Qin, Haitang Yue, Zhuan Bian, Miao He

**Affiliations:** ^1^ State Key Laboratory Breeding Base of Basic Science of Stomatology (Hubei‐MOST) and Key Laboratory of Oral Biomedicine Ministry of Education School & Hospital of Stomatology Wuhan University Wuhan China; ^2^ Key Laboratory of Analytical Chemistry for Biology and Medicine (Ministry of Education) College of Chemistry and Molecular Sciences State Key Laboratory of Virology, Wuhan University Wuhan China

**Keywords:** Chinese Han, Interferon Regulatory Factor 6, sequencing study, Van der Woude syndrome

## Abstract

**Background:**

Interferon Regulatory Factor 6 (*IRF6*) gene encodes a member of the IRF family of transcription factors. Mutations in *IRF6* cause Van der Woude Syndrome (VWS), which is the most common malformation of syndromic orofacial clefts in humans.

**Methods:**

Here, we performed sequencing studies of six families with VWS in the Chinese Han population. The entire *IRF6*‐coding region and the exon–intron boundaries including exons 3–8 and part of exon 9 were screened among all the collected family members by Sanger sequencing.

**Results:**

We found a novel splice site variant c.175‐6T>A, two novel missense variants (p.Lys66Arg and p.Pro107Thr), in addition with a previously reported missense variant (p.Leu87Phe), which were all located in and nearby exon 4 of *IRF6*. Meanwhile, a novel frameshift variant p.G257Vfs*46 in exon 7 of *IRF6* was also detected. All the mutations presented to be co‐segregated in each family.

**Conclusion:**

Our study has advanced the understanding of the genetic architecture of VWS and provides the basis for genetic counseling, antenatal diagnosis, and gene therapy of high risk groups.

## Graphical abstract

It is a study on *IRF6* sequencing of six Van der Woude Syndrome families in the Chinese Han population. And four novel variants were detected in the study.

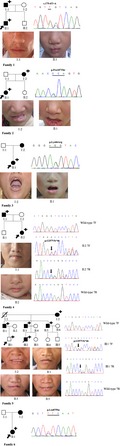

## 1 INTRODUCTION

Orofacial clefts (OFC) are the most common craniofacial malformations in humans with complex pathogenesis, which are often classified as syndromic or nonsyndromic (Dixon, Marazita, Beaty, & Murray, 2011; Mossey, Little, Munger, Dixon, & Shaw, 2009). So far, according to the Online Mendelian Inheritance in Man (OMIM) database (http://www.omim.org), more than 500 syndromes with clefting phenotypes have been reported worldwide. Among them, VWS (OMIM: 119300) is the most common form, accounting for 2% of all OFC cases, with the birth prevalence rate of 1 in 35,000 approximately (Leslie et al., 2013). The VWS is inherited as an autosomal dominant mode with high penetrance (96.7%) and variable manifestations (Janku et al., 1980; de Lima et al., 2009). In VWS patients, lower lip pits and/or sinuses are considered to be the main characteristic features, which varies from a single barely evident depression to bilateral fistulae. Lip pits totally present in over 80% of VWS cases, and sometimes are the only visible defect in 64% VWS patients. The other frequently associated phenotypes with VWS include cleft lip with or without cleft palate (CL/P), cleft palate only (CPO), and hypodontia (Rizos & Spyropoulos, 2004; Wang et al., 2019).

*IRF6* (OMIM: 607199) has been reported to cause VWS in almost 70% of the affected families among different populations (Peyrard‐Janvid et al., 2014). IRF6, which belongs to a family of transcription factors, has two conserved domains, a winged‐helix DNA‐binding domain (DBD), and a protein‐binding domain which is also termed SMIR (Smad‐interferon regulatory factor‐binding domain; Kondo et al., 2002; Leslie et al., 2013; de Lima et al., 2009). To date, over 300 different mutations in *IRF6*, including missense, nonsense, frameshift, microdeletions, splice‐site mutations, and so forth, have been reported to cause VWS, Popliteal pterygium syndrome (PPS, MIM: 119500), Oral clefts and other diseases, of which more than 200 mutations were detected in VWS. A majority of these identified mutations are nonrandomly distributed and observed to be enriched in either exon 3 and 4, which encodes the DNA‐binding domain, or in exon 7 and 9, which are in the protein‐binding domain (Busche, Hehr, Sieg, & Gillessen‐Kaesbach, 2016; Charzewska et al., 2015; Kondo et al., 2002; Leslie et al., 2013; de Lima et al., 2009).

To gain a better understanding of the genetic basis underlying VWS in the Chinese Han population, we screened the genomic DNA from six Chinese pedigrees with VWS by Sanger sequencing of all coding exons and the flanking intronic regions in *IRF6* (exons 3–9). We found a novel splice site variant c.175‐6T>A, two novel missense variants (p.Lys66Arg and p.Pro107Thr), and a previously reported missense variant (p.Leu87Phe), which were all in exon 4. In addition, a novel frameshift variant p.G257Vfs*46 in exon 7 was also detected.

## 2 METHODS

### 2.1 Ethical compliance and samples

The current study was approved by the institutional ethics committee of the Hospital of Stomatology, Wuhan University. All the patients were interviewed and clinically assessed by at least two experienced clinicians, and a detailed questionnaire was completed to clarify the diagnosis and understand the genetic background. The criteria for the diagnosis of VWS were the presence of lower lip pits and/or sinuses, with or without CL/P or CPO. Informed written consent was taken from each proband or the guardians of those who were under age. Peripheral blood samples were collected from affected probands, their parents, and from available affected and unaffected family members.

Approximately 4 ml EDTA anticoagulated venous blood sample was collected from each participant. Genomic DNAs of the cases were extracted from peripheral blood lymphocytes using the standard sodium dodecyl sulfate‐proteinase K‐phenol/chloroform method. After quality control, DNAs were diluted to working concentrations of 100 ng/μl for sequencing. DNA samples of 200 unrelated healthy controls were selected from our previous study.

### 2.2 Clinical information

**Figure 1:**
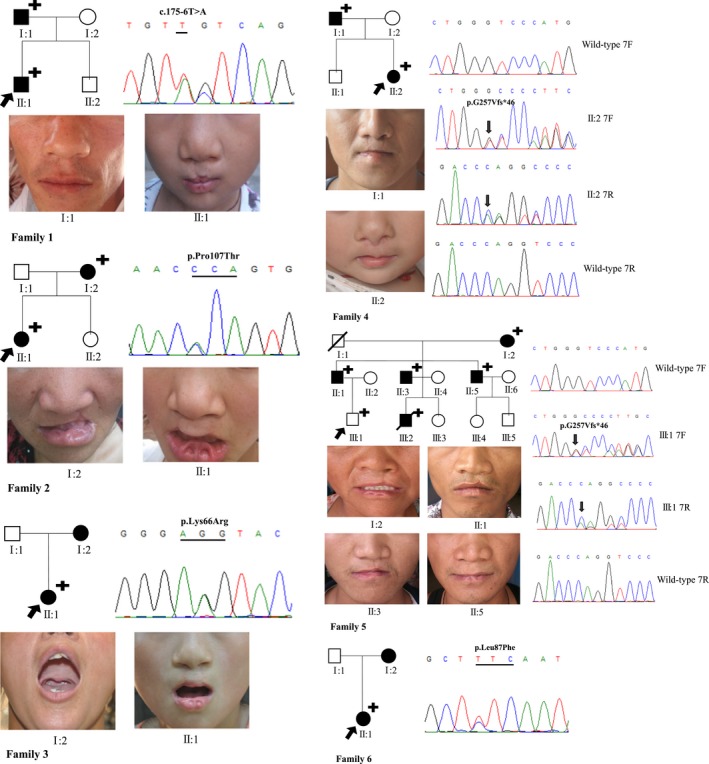
Pedigrees of the Van der Woude Syndrome (VWS) families. The black arrows point to patient in each VWS family. The plus signs represent the patients who have bilateral lower lip pits. The solid black circle and square represent the patients who have CL/P. In Family 1, a novel splice site variant c.175‐6T>A was found in Ⅰ:1 and Ⅱ:1. Two novel missense variants p.Pro107Thr and p.Lys66Arg were detected in Family 2 and Family 3, respectively. A novel frameshift mutation p.G257Vfs*46 was found in both Family 4 and Family 5, and affected totally seven patients. In Family 6, a previously reported missense variant p.Leu87Phe was detected in Ⅰ:2 and Ⅱ:1

The six enrolled families were listed as follows (Figure 1; Table S1).

Family 1: Patient Ⅱ:1 had bilateral lower lip pits, and complete bilateral cleft of the lip, palate and alveolar (BCCLP). The patient's father had bilateral lower lip pits and incomplete right cleft of the lip (RICL).

Family 2: Patient Ⅱ:1 had bilateral lower lip pits and BCCLP. The patient's mother had bilateral lower lip pits, and complete left cleft of the lip, palate, and alveolar (LCCLP).

Family 3: Patient Ⅱ:1 had bilateral lower lip pits, complete bilateral cleft of the lip (BCCL), incomplete cleft palate (ICP), and complete bilateral cleft of the alveolar (BCCA). The patient's mother had ICP.

Family 4: Patient Ⅱ:2 had bilateral lower lip pits, BCCL and BCCA. The patient's father also had bilateral lower lip pits, BCCL and BCCA.

Family 5: Patient Ⅲ:1 had bilateral lower lip pits without CL/P (No image data). The patient's grandmother, father, and his first uncle all had bilateral lower lip pits, complete right cleft of the lip (RCCL), and complete right cleft of the alveolar (RCCA). In addition, the patient's second uncle had bilateral lower lip pits, complete left cleft of the lip (LCCL), and complete left cleft of the alveolar (LCCA).

Family 6: Patient Ⅱ:1 had bilateral lower lip pits, and complete right cleft of the lip, palate and alveolar (RCCLP). The patient's mother had RCCLP (No image data).

### 2.3 Sequencing

Exons 3–8 and part of exon 9 of *IRF6* which spanned the entire *IRF6*‐coding region and the exon–intron boundaries were amplified by using the primers that were designed via Primer3 (http://primer3.ut.ee/; Rozen & Skaletsky, 2000; Untergasser et al., 2012). The polymerase chain reaction (PCR) primers were made by Sangon Biotech Co., Ltd. The sequence of the primers are shown in Table S2. After amplifying and purifying, the DNA sequences were detected with an ABI 3730XL genetic analyzer (Applied Biosystems). Sequence aligning and analyzing were performed by CHROMAS and BLAST program on the National Center for Biotechnology Information (NCBI). To confirm the DNA sequence variants, sequencing of the opposite strand was performed. In addition, to exclude the possibility of single‐nucleotide polymorphism (SNP), 200 unrelated healthy controls were examined, respectively. After these, we then compared the mutations found in this study to mutations which are shown in Ensembl database (http://asia.ensembl.org/index.html) and the Human Gene Mutation Database (HGMD, http://www.hgmd.cf.ac.uk/ac/index.php). Reference sequences for *IRF6* genomic DNA, cDNA, and protein were NG_007081.2, NM_006147.4, and NP_006138.1, respectively.

To predict the functional effects of the mutations identified in the study, in silico prediction programs were conducted by using bioinformatics tools, including the Human Splicing Finder (HSF v.3.1, http://www.umd.be/HSF; Desmet et al., 2009), Protein Variation Effect Analyzer (PROVEAN, http://provean.jcvi.org/index.php; Choi & Chan, 2015), PolyPhen‐2 (http://genetics.bwh.harvard.edu/pph2/; Adzhubei et al., 2010), Mutation Taster (http://www.mutationtaster.org/; Schwarz, Cooper, Schuelke, & Seelow, 2014) and HOPE (https://www3.cmbi.umcn.nl/hope; Venselaar, Beek, Kuipers, Hekkelman, & Vriend, 2010).

## 3 RESULTS

All the six pedigrees involving two or three generations were continuously inheriting, that was at least one patient affected in each generation. Patients all had bilateral lower lip pits and CL/P except patient in Family 5 who had only bilateral lower lip pits, but the phenotypes among them varied severity. The affected parents also had bilateral lower lip pits and CL/P, except the affected mothers in Family 3 and 6 who did not show lip pits/sinuses. It was worth mentioning that in Family 1 to Family 3, and Family 6 the clinical phenotypes of the offsprings were more serious than their affected parents (Figure 1).

According to the sequencing analyses, we found a novel frameshift variant, two novel and one previously reported missense variants, as well as a novel splice variant, which were all heterozygous mutations (Figure 1; Figure S1). None of the novel mutations was detected in healthy controls or previously reported. In Family 1, a new splice variant c.175‐6T>A was found at the splice site of exon 4. In Family 2, a novel missense variant c.319C>A, led to a p.Pro107Thr change in amino acid level. In Family 3, we found a new missense variant c.197A>G at a previously reported mutation site, that led to p.Lys66Arg which was different from previously reported p.Lys66X in VWS (Butali et al., 2014) and p.Lys66Thr found in PPS (Kondo et al., 2002). A previously known missense variant c.259C>T (p.Leu87Phe; de Lima et al., 2009) was found in Family 6. All the above mutations were in and nearby exon 4. In addition, a single‐base deletion in exon 7, c.770delG leading to a frameshift and introducing a premature stop codon p.G257Vfs*46 was identified in Family 4 and Family 5. The mutations presented to be co‐segregated in each family.

**Figure 2:**
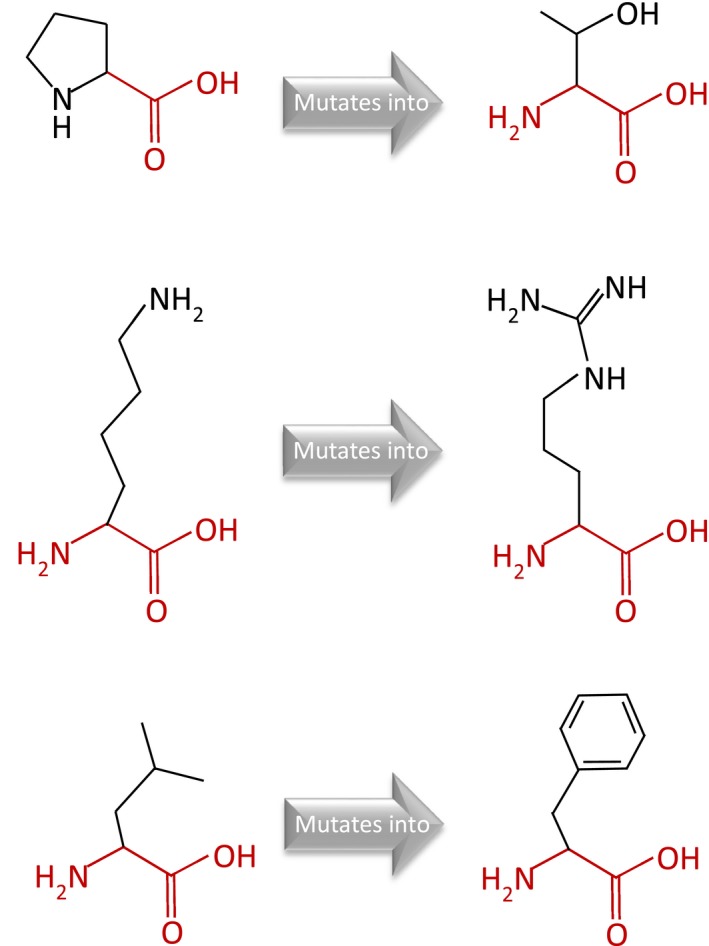
Schematic structures of the original (left) and the mutant (right) amino acid. The backbone, which is the same for each amino acid, is colored red. The side chain, unique for each amino acid, is colored black. (a) A Proline into a Threonine at position 107 in Van der Woude Syndrome (VWS) Family 2; (b) a Lysine into an Arginine at position 66 in VWS Family 3; (c) a Leucine into a Phenylalanine at position 87 in VWS Family 6

**Figure 3:**
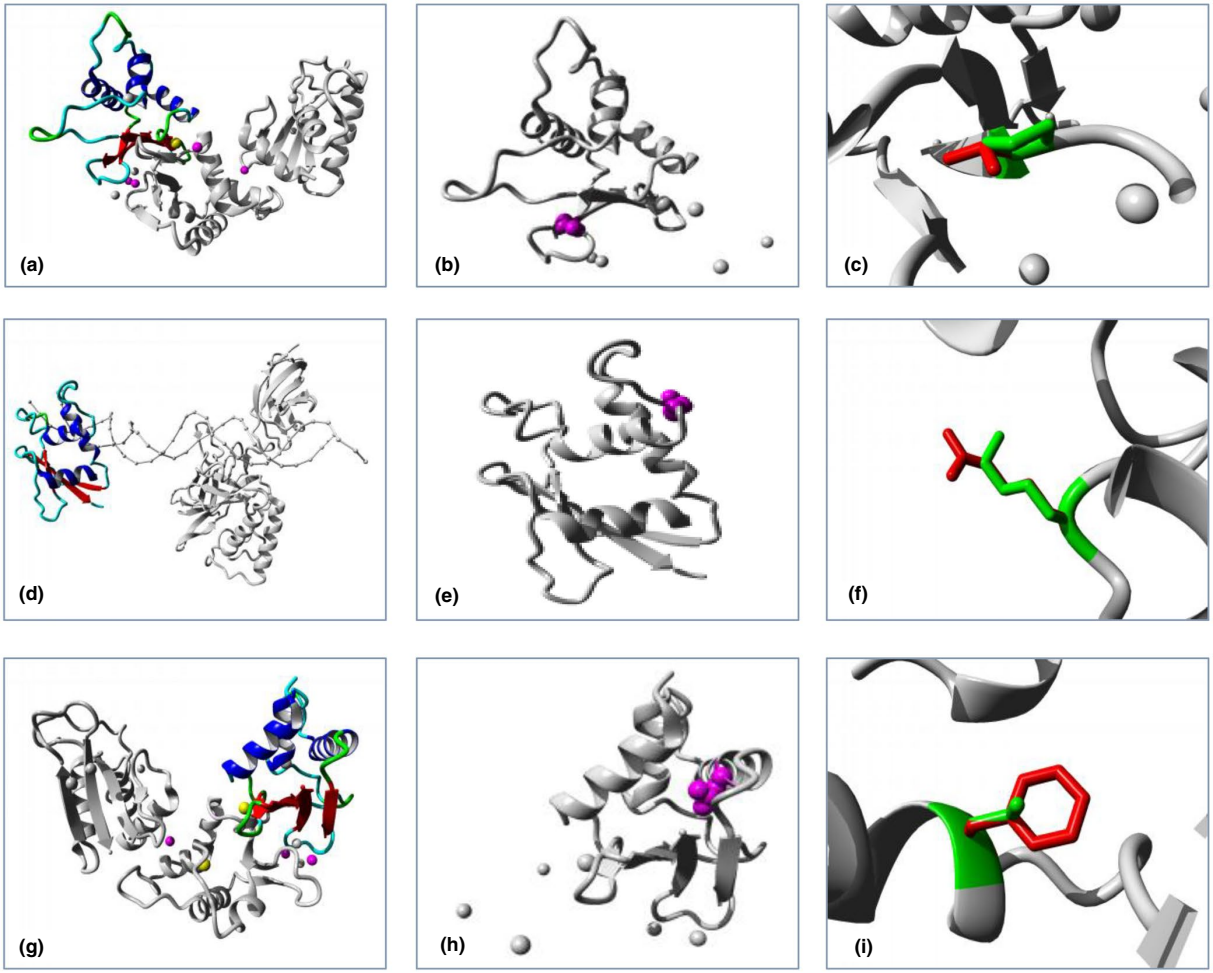
Overview of the IRF6 protein in ribbon presentation. The protein is colored by element; α‐helix = blue, β‐strand = red, turn = green, 3/10 helix = yellow and random coil = cyan. Other molecules in the complex are colored gray when present (a, d and g). The protein is colored gray, the side chain of the mutated is colored magenta and shown as small balls (b, e and h). The side chains of both the wild‐type and the mutant residue are shown and colored green and red, respectively (c, f and i). (b, e and h) are the partial enlarged views of (a, d and g). (c, f and i) are close‐ups of the mutations at positions 107, 66, and 87, respectively

The three missense mutations were all predicted to be destructive according to in silico studies (Table 1). HOPE revealed that at the amino acid position 107, the wild‐type residue was more hydrophobic than the mutant residue, which changed from nonpolar amino acid to neutral polar amino acid, while at the position 66 and 87 the hydrophobicity value did not change between residues of the wild type and the mutant. The mutant residue is bigger than the wild‐type residue at positions 66 and 87 (Figure 2a–c). The three amino acids were all located in the DNA‐binding domain of *IRF6*. In the 3D‐structure, it can be seen that at position 87 the wild‐type residue was located in an α‐helix. The mutation converted the wild‐type residue in a residue that did not prefer α‐helices as the secondary structure. Of the other two amino acids that were both located at the random coil regions (Figure 3a–i; Figures S2–S7), the wild‐type residue at position 66 was annotated to be involved in DNA interaction type: "IRF tryptophan pentad repeat". The three missense mutations changed the size, hydrophobicity, and structure of each amino acid residue, thus affecting the function and disturbed regulation of the protein.

**Table 1:** *IRF6* variants in Van der Woude Syndrome syndrome families in this study

Families	Proband	Gender	Mutation	Variant cDNA[Link]	Amino acid change[Link]	Inheritance	Protein domain	PROVEAN (Class; Score)	SIFT (Class; Score)	PolyPhen (Class; Score)	Mutation Taster	Exon
1	Ⅱ:1	M	—	c.175−6T>A	—	co‐segregation	—	—	—	—	—	Splice site (4)
2	Ⅱ:1	F	missense	c.319C>A	p.Pro107Thr	co‐segregation	DBD	Deleterious; −7.07	Damaging; 0.0	probably damaging; 1	disease causing	4
3	Ⅱ:1	F	missense	c.197A>G	p.Lys66Arg	co‐segregation	DBD	Deleterious; −2.68	Damaging; 0.0	probably damaging; 0.994	disease causing	4
4	Ⅱ:2	F	frameshift	c.770delG	p.G257Vfs*46	co‐segregation	SMIR	—	—	—	disease causing	7
5	Ⅲ:1	M	frameshift	c.770delG	p.G257Vfs*46	co‐segregation	SMIR	—	—	—	disease causing	7
6	Ⅱ:1	F	missense	c.259C>T	p.Leu87Phe*	co‐segregation	DBD	Deleterious; −3.61	Damaging; 0.0	probably damaging; 1	disease causing	4

NM_006147.4.

NP_006138.1.

a This variant has been previously identified (de Lima et al., 2009).

## 4 DISCUSSION

Phenotypic heterogeneity are detected in the six VWS families. Two of the fifteen patients do not show the typical phenotypes: lip pits/sinuses, which are consistent with previous findings (Janku et al., 1980; Kaul, Mahajan, Gupta, & Kotwal, 2014). It was reported that sometimes the lip pits may be too tiny to be found, such as the form of a single barely evident depression or transverse slit (Desmyter et al., 2010; Rintala & Ranta, 1981). So careful examinations are needed when a patient has the family history of orofacial clefting. The main cause of lip pits present in VWS is thought to be notching of the lip with fixation of the tissue at the base of the notch or failure of a complete union of the embryonic lateral sulci of the lip, at an early stage of the labial development (Deshmukh et al., 2014; Ural, Bilgen, Cakmakli, & Bekerecioglu, 2019). The phenotypes of both lip pits and CL/P show variable forms, even in the same family the manifestations of the patients are different though they carry the same mutation in *IRF6*, which is in accordance with previous studies (Butali et al., 2014; de Lima et al., 2009; Tan, Lim, Lim, & Lee, 2017), and the reason may be epigenetic modification of DNA in *IRF6*. By comparing the phenotypes and genotypes among the six VWS families, we observed that the type of mutation has no correlation in predicting the severity of this disease, which was similarly described in a previous study (Butali et al., 2014).

Totally, five different mutations of *IRF6* in six Chinese Han VWS families were identified. We found a novel splice‐site variant c.175‐6T>A at 5' end of exon 4, which is predicted to locate in an acceptor site by using the HSF database. Although no significant splicing motif alteration was detected by searching c.175‐6T>A or c.175‐5C>G (a SNP, rs7552506), respectively, in HSF database, when searching the combination of them which presented in both patient and his affected father in Family 1 (Figure 1), it is predicted to alter the wild‐type acceptor site and most probably affect splicing. The novel frameshift mutation c.770delG (p.G257Vfs*46) found in exon 7 is predicted to result in a change in the *IRF6* reading frame and introduce a stop codon within exon 7, leading to 166 amino acids truncation of the protein encoded by *IRF6*, thus destroying the function of *IRF6*. The frameshift mutation was predicted to be disease causing according to the Mutation Taster database.

All the three missense mutations (p.Pro107Thr, p.Lys66Arg, and p.Leu87Phe) are localized in regions encoding the DNA‐binding domain. At the amino acid 107 position, HOPE revealed that the novel mutated residue was not in contact with a metal, however, one of the neighboring residues did make a metal‐contact that might be affected by the mutation in its vicinity. And the mutation might cause loss of hydrophobic interactions with other molecules on the surface of the protein. The wild‐type residue proline is known to be very rigid and therefore induce a special backbone conformation which might be required at this position, but the mutation can disturb this special conformation. At position 66, a mutation to "Threonine" was found at this position and was annotated as PPS (Kondo et al., 2002) and a nonsense variant p.Lys66X was reported in a VWS family in sub‐Saharan Africa (Butali et al., 2014), which are different from the mutation found in our study. HOPE showed that the residue was located on the surface of the protein, and mutation of this residue could disturb interactions with other molecules or other parts of the protein. Based on these studies, we speculate that the amino acid in position 66 in *IRF6* is a mutational hot spot of syndromic CL/P among different populations. The same mutation p.Leu87Phe found in our study was previously reported by de Lima et al. in the VWS family (de Lima et al., 2009). Of the three missense mutations, the mutated residues are located in a domain that is important for binding of other molecules and in contact with residues in a domain that is also important for binding. The mutations might disturb the interaction between these two domains and thereby affect the function of the protein. None of the mutations at these three positions was observed in other homologous sequences. Based on the in silico studies such as PROVEAN, SIFT, PolyPhen and Mutation Taster, the three missense mutations were all predicted to be disease causing.

The five mutations in our study segregate in their families, respectively. These patterns of inheritance are in accordance with the pattern of VWS caused by autosomal dominant inheritance. As the most common form of syndromic OFC, VWS affects about 200,000 people in the world. However, the genetic basis of VWS is still not clear. More sequencing and functional investigations of VWS would provide theoretical basis for genetic consultation, antenatal diagnosis, and gene therapy of CL/P. Meanwhile, it is helpful to elucidate the pathogenesis of nonsyndromic CL/P.

## CONFLICT OF INTEREST

The authors have no conflicts of interest relevant to this article.

## AUTHOR CONTRIBUTIONS

Z. B. and Y. Q. Y. conceived the study. Y. Q. Y. and Y. T. W. performed the experiments, analyzed the data, and wrote the manuscript. C. Q. Q. collected the samples. M. H. and H. T. Y. participated in the study design. M. H., C. Q. Q., and H. T. Y. reviewed and revised the manuscript. All authors approved the final manuscript as submitted and agree to be accountable for all aspects of the work.
