# Supplementary material for: Novel IRF6 mutations in Chinese Han families with Van der Woude syndrome
Source: Mol Genet Genomic Med. 2020 Feb 28;8(5):e1196. doi: 10.1002/mgg3.1196 (PMC7216816; doi:10.1002/mgg3.1196)
Supplement: Supplementary file 1 [file MGG3-8-e1196-s001.docx]

Ⅰ:1

Ⅰ:2

Ⅱ:1

Ⅱ:2


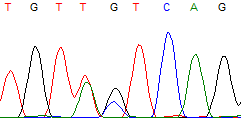


c.175-6T>A

Ⅰ:1


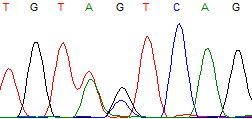


c.175-6T>A

Ⅱ:1

**Family 1**


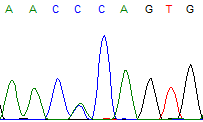


p.Pro107Thr

Ⅰ:2

Ⅰ:1

Ⅰ:2

Ⅱ:1

Ⅱ:2


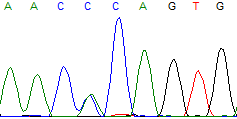


p.Pro107Thr

Ⅱ:1

**Family 2**


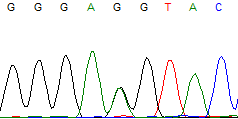


p.Lys66Arg

Ⅱ:1


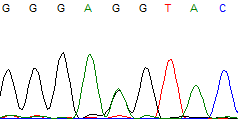


p.Lys66Arg

Ⅰ:2

Ⅰ:1

Ⅰ:2

Ⅱ:1

**Family 3**

Ⅰ:1

Ⅰ:2

Ⅱ:1

Ⅱ:2


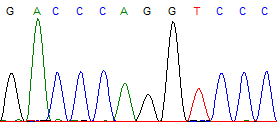


Ⅰ:2

7R


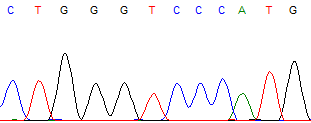


Ⅰ:2

7F


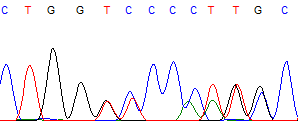


p.G257Vfs*46

7F

Ⅰ:2


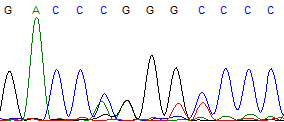


7R

Ⅰ:2


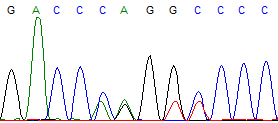


Ⅰ:1

7R


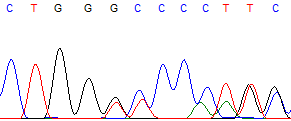


p.G257Vfs*46

Ⅰ:1

7F


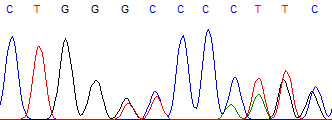


p.G257Vfs*46

Ⅱ:2

7F


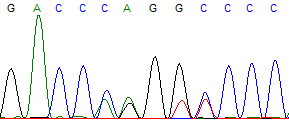


Ⅱ:2

7R

**Family 4**

Ⅰ:1

Ⅰ:2

Ⅱ:1

Ⅱ:2

Ⅲ:1

Ⅱ:3

Ⅱ:4

Ⅲ:2

Ⅱ:5

Ⅱ:6

Ⅲ:4

Ⅲ:3

Ⅲ:5


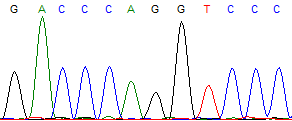


7R

Ⅱ:2


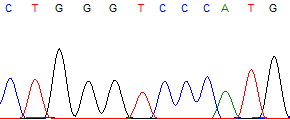


7F

Ⅱ:2


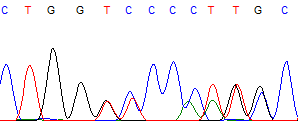


p.G257Vfs*46

7F

Ⅰ:2


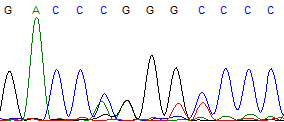


7R

Ⅰ:2


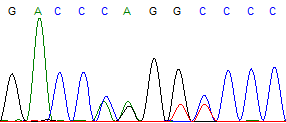


7R

Ⅱ:1


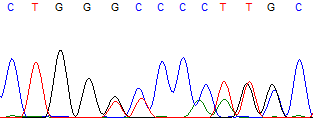


p.G257Vfs*46

7F

Ⅱ:1


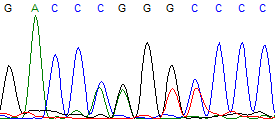


7R

Ⅱ:3


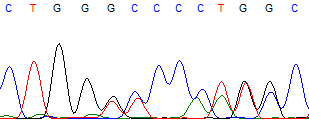


p.G257Vfs*46

7F

Ⅱ:3

7F


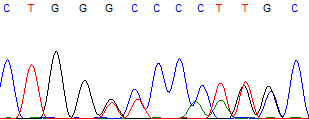


p.G257Vfs*46

Ⅱ:5

7F


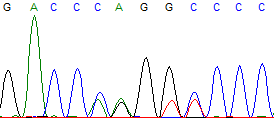


7R

Ⅱ:5


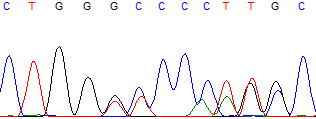


p.G257Vfs*46

7F

Ⅲ:1


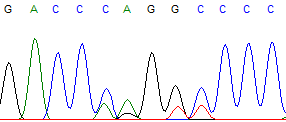


7R

Ⅲ:1

**Family 5**


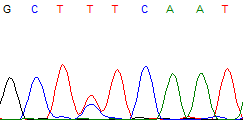


p.Leu87Phe

Ⅱ:1


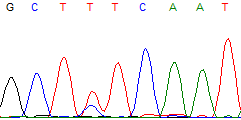


p.Leu87Phe

Ⅰ:2

Ⅰ:1

Ⅰ:2

Ⅱ:1

**Family 6**

**Supplementary Figure 1.** **Chromatograms of *IRF6* gene mutations in the six VWS syndrome families.** The black arrows point to the proband in each VWS family. The plus signs represent the patients who have bilateral lower lip pits. The solid black circle and square represent the patients who have CL/P.
