# Supplementary figures and images for: Novel IRF6 mutations in Chinese Han families with Van der Woude syndrome
Source: Mol Genet Genomic Med. 2020 Feb 28;8(5):e1196. doi: 10.1002/mgg3.1196 (PMC7216816; doi:10.1002/mgg3.1196)

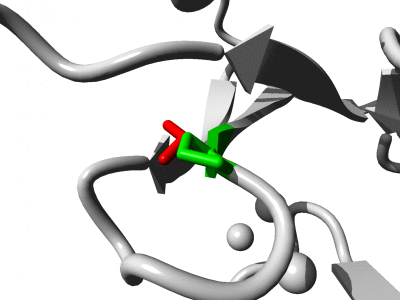

Supplement: Supplementary file 2 [file MGG3-8-e1196-s002.gif]

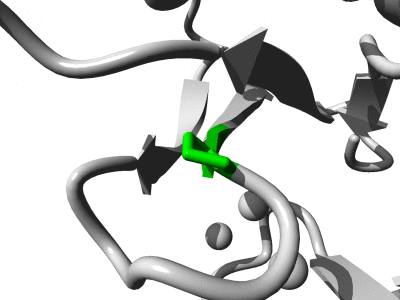

Supplement: Supplementary file 3 [file MGG3-8-e1196-s003.gif]

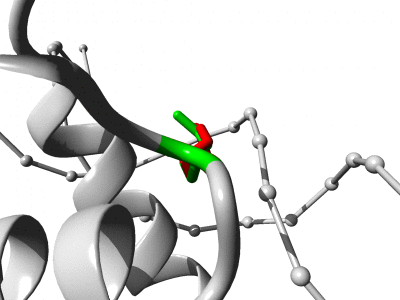

Supplement: Supplementary file 4 [file MGG3-8-e1196-s004.gif]

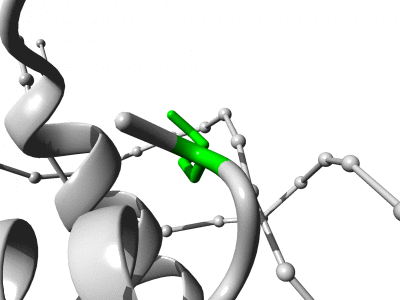

Supplement: Supplementary file 5 [file MGG3-8-e1196-s005.gif]

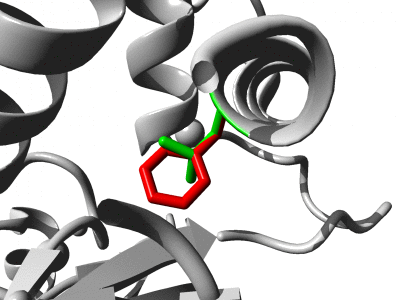

Supplement: Supplementary file 6 [file MGG3-8-e1196-s006.gif]

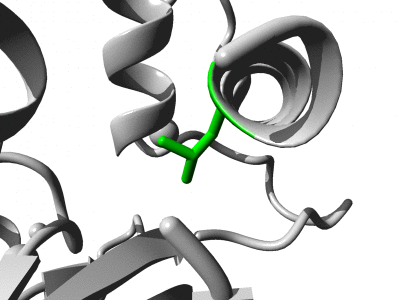

Supplement: Supplementary file 7 [file MGG3-8-e1196-s007.gif]
